# Persistent *STAG2* mutation despite multimodal therapy in recurrent pediatric glioblastoma

**DOI:** 10.1038/s41525-020-0130-7

**Published:** 2020-06-01

**Authors:** Christopher S. Hong, Juan C. Vasquez, Adam J. Kundishora, Aladine A. Elsamadicy, Jason M. Beckta, Amrita Sule, Asher M. Marks, Nalin Leelatian, Anita Huttner, Ranjit S. Bindra, Michael L. DiLuna, Kristopher T. Kahle, E. Zeynep Erson-Omay

**Affiliations:** 1 Department of Neurosurgery, Yale School of Medicine, New Haven, CT 06511 USA; 2 Department of Pediatrics, Yale School of Medicine, New Haven, CT 06511 USA; 3 Department of Therapeutic Radiology, Yale School of Medicine, New Haven, CT 06511 USA; 4 Department of Pathology, Yale School of Medicine, New Haven, CT 06511 USA

**Keywords:** Cancer genetics, Genetics research

## Abstract

Similar to their adult counterparts, the prognosis for pediatric patients with high-grade gliomas remains poor. At time of recurrence, treatment options are limited and remain without consensus. This report describes the genetic findings, obtained from whole-exome sequencing of a pediatric patient with glioblastoma who underwent multiple surgical resections and treatment with standard chemoradiation, as well as a novel recombinant poliovirus vaccine therapy. Strikingly, despite the variety of treatments, there was persistence of a tumor clone, characterized by a deleterious *STAG2* mutation, whose deficiency in preclinical studies can cause aneuploidy and aberrant mitotic progression, but remains understudied in the clinical setting. There was near elimination of an *EGFR* mutated and amplified tumor clone after gross total resection, standard chemoradiation, and poliovirus therapy, followed by the emergence of a persistently *STAG2* mutated clone, with rare mutations in *PTPN11* and *BRAF*, the latter composed of a novel deleterious mutation previously not reported in pediatric glioblastoma (p.D594G). This was accompanied by a mutation signature shift towards one characterized by increased DNA damage repair defects, consistent with the known underlying *STAG2* deficiency. As such, this case represents a novel report following the clinical and genetic progression of a *STAG2* mutated glioblastoma, including treatment with a novel and emerging immunotherapy. Although *STAG2* deficiency comprises only a small subset of gliomas, this case adds clinical evidence to existing preclinical data supporting a role for *STAG2* mutations in gliomagenesis and resistance to standard therapies.

## Introduction

High-grade gliomas in the pediatric population represent approximately 6.5% of all newly diagnosed childhood brain tumors^1^. Similar to adults, prognosis remains poor with 5-year survival rates under 20%^2^. Standard of care therapy is comprised of maximal safe surgical resection followed by adjuvant temozolomide and radiation therapy (RT), based on the Stupp protocol originally established in the adult setting^3^. Despite prior clinical trials showing varying degrees of therapeutic efficacy, there are no standard treatment options at time of recurrence^4^.

Stromal Antigen-2 (*STAG2*) encodes a subunit within the cohesin complex, whose inactivation has been shown to cause aneuploidy through sister chromatid cohesion, and increased DNA damage that may promote further mutagenesis^5,6^. While *STAG2* deficiency characterizes a small subset of glioblastoma, increasing preclinical evidence supports a driver role of this mutation in tumor formation and resistance to standard therapies in isogenic STAG2 deficient cell lines^7,8^.

**Fig. 1:**
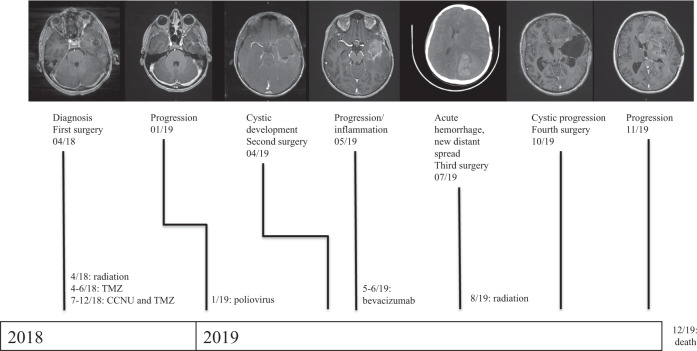
Clinical progression of the index case. A timeline of the patient's clinical course is shown with representative imaging at the top and corresponding medical and radiation treatments below.

In this report, we describe the genetic evolution of a glioblastoma in a pediatric patient who underwent standard chemoradiation followed by recombinant poliovirus therapy as a part of his treatment regimen (Fig. 1). Whole-exome sequencing (WES) of surgically resected specimens at time of diagnosis and subsequent multiple recurrences revealed striking persistence of the *STAG2* mutation, accompanied by a shift towards a mutational signature that is seen in cancers with DNA repair defects, and the emergence of new secondary mutations, including an inactivating, non-V600E (i.e., class III) *BRAF* mutation^9^, previously not reported in pediatric glioma.

## Results

### Case description

A 14-year-old male with no past medical history was brought to an outside hospital with an acute episode of confusion and nonsensical speech in the context of progressively worsening headaches. Computed tomography (CT) demonstrated a left temporal parenchymal hematoma with surrounding edema effacing the temporal horn. He suffered a generalized seizure necessitating intubation and was transferred to our institution for further care. Magnetic resonance imaging (MRI) with MR angiography showed a 2.2 cm enhancing predominantly cystic lesion adjacent to the hematoma and within the left anterior temporal pole, concerning for an underlying neoplasm (Supplementary Fig. 1a, b). He was subsequently extubated and underwent functional MRI to localize language function, which did not reveal tumor involvement of the language area, prior to resection of the lesion. Intraoperatively, a well-demarcated plane was observed between the tumor capsule and edematous white matter, and a gross total resection (GTR) was achieved. Final pathology was consistent with WHO grade IV glioblastoma (Supplementary Fig. 2a) with a Ki67% index over 20%. Routine genetic and molecular analyses demonstrated IDH1 and ATRX wild-type status, intact 1p/19q chromosomal arms, and partial MGMT methylation. The patient recovered uneventfully from surgery and underwent adjuvant RT (5940 cGy delivered in 33 fractions) with concurrent temozolomide (90 mg/m^2^).

After completion of chemoradiation, surveillance imaging obtained 3 months after surgery showed progressive FLAIR abnormalities along the anterior left temporal lobe, albeit without new nodular enhancement, concerning for progression. Subsequently, the decision was made to give maintenance chemotherapy with lomustine and temozolomide per ACNS0423^10^. Nine months after surgery and four cycles of maintenance chemotherapy, repeat imaging demonstrated new nodular enhancement within the inferior aspect of the resection bed with mild surrounding FLAIR abnormalities (Supplementary Fig. 1c, d). At this point, he was referred to an outside institution for consideration of immunotherapy trials, and he subsequently underwent recombinant poliovirus therapy via convection-enhanced delivery. Subsequent surveillance imaging did not demonstrate regression of enhancing disease.

Three months after receiving the poliovirus, he presented with acute worsening of headaches that had progressed over the past few weeks despite an otherwise neurologically intact exam. Repeat MRI showed an increased size of the rim-enhancing mass measuring up to 5.3 cm with a prominent cystic component (Supplementary Fig. 1e, f). After multidisciplinary discussion including from the outside institution where he underwent poliovirus therapy, the patient underwent repeat surgery for cyst decompression and GTR of the solid superior and posterior enhancing nodules along the cyst wall. Pathology re-demonstrated recurrent glioblastoma (Supplementary Fig. 2b) with marked absence of any immune inflammatory response (Supplementary Fig. 3). Postoperatively, he recovered uneventfully but within weeks after surgery developed worsening headaches accompanied by radiographic evidence of increased peri-tumoral edema, which improved with corticosteroid therapy. Subsequently, the decision was made to initiate bevacizumab therapy (7.5 mg/kg every 3 weeks) to treat the inflammation, and to facilitate steroid taper. However, on repeat imaging 2 months after starting bevacizumab therapy, there was evidence of a recurrent enhancement within the resection bed, as well as a new focus of enhancement within the occipital lobe with signs of internal hemorrhage, prompting cessation of bevacizumab therapy (Supplementary Fig. 1g, h). Within days of stopping treatment, the patient became acutely obtunded and was found to have significant intratumoral hemorrhages within the temporal and occipital lobes (Supplementary Fig. 1i, j), requiring emergent decompressive hemicraniectomy and hematoma evacuation. Pathology showed areas of viable tumor within largely hemorrhagic material (Supplementary Fig. 2c). He was discharged to a rehabilitation center with significant right-sided hemiplegia.

One month after surgery, the patient underwent radiation (3500 cGy delivered in ten fractions) to the new occipital focus, as well as the previous temporal bed, but he continued to suffer persistent headaches. Repeat imaging one month after completing RT showed a growing left temporal tumor-related cyst, and due to the severity of his symptoms, he underwent a fourth surgery for left temporal lobectomy and tumor cyst fenestration into the basal cisterns with histopathological confirmation of progressive tumor (Supplementary Fig. 2d). Within days after discharge, he re-presented with concern for cerebrospinal fluid leakage from his incision. This was managed conservatively with lumbar drainage, but his hospital course was significant for repeat surveillance imaging demonstrating significant growth of the tumor from the temporal focus, crossing over the corpus callosum into the contralateral hemisphere (Supplementary Fig. 1k, l). After extensive discussions, the decision was made to cease further invasive interventions, and the patient went home with hospice services. He passed away within one month after discharge.

### Genomic analysis

**Fig. 2:**
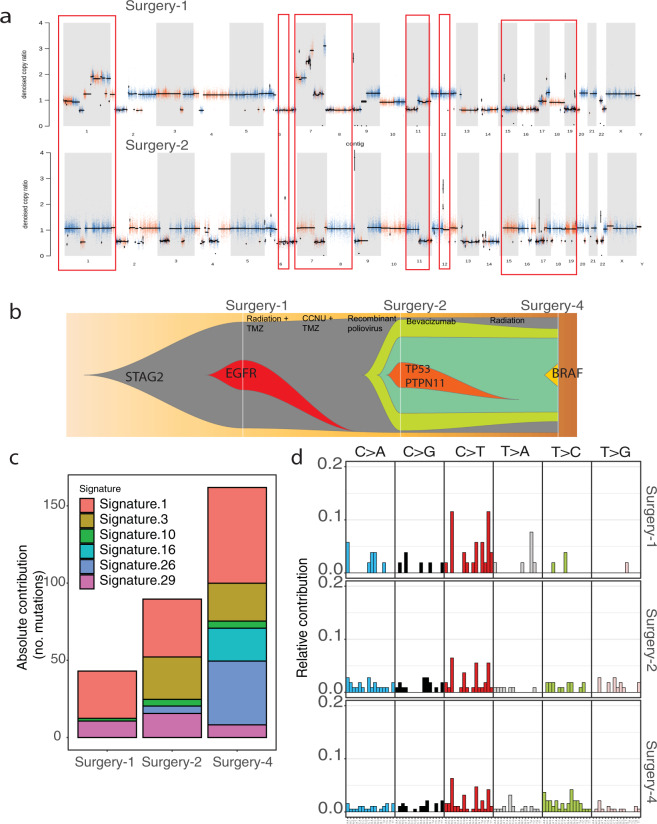
Genomic analysis. **a** CNV of the tumor from the first and second surgeries, in the upper and lower panel respectively, depict the loss of amplification on chromosome 7, beside other events. **b** Representation of clonal evolution of tumors from three distinct surgeries using fishplot^46^, where the width of each different colored clone represents clonal fraction at the given timepoint, in relationship to the patient’s surgeries and treatments. **c** Mutation signatures of three specimens represent the most aberrant COSMIC signatures. **d** Distribution of 96 mutation signatures of all specimens.

WES was performed on tumor samples from the first, second, and the fourth surgeries, together with matching normal blood to identify somatic single nucleotide variations (SNV), insertion/deletions (INDEL), and copy-number variations (CNV). Inadequate specimen was available from the third surgery (emergent decompressive hemicraniectomy) for genetic analysis. Tumor purities for these 3 tumors were computationally predicted as 98.5%, 97.5%, and 73.8%, for specimen from surgery 1, surgery 2, and surgery 4, respectively. The CNV profile for the specimens from the second and fourth surgeries were similar, while specimens from the first surgery harbored additional alterations including large scale amplification on chromosome 7, including the amplification of EGFR, that was lost prior to the second surgery (Fig. 2a). Other CNV events, such as the amplification on chromosome 1q, deletion of chromosomes 8,11p, 15, 16, and 19 were lost after the first tumor resection. There was also emergence of other CNV events in the specimen from second surgery, such as deletions on chromosomes 3, 4, 20, and 22 as well as amplifications on chromosomes 6 (focal), 11q and 12 (focal) depicting the complex evolution of the tumor under the progression and treatment pressure. Interestingly, focal amplification on chromosome 12 overlapped with CDK4 gene, which was previously reported to be amplified with high ploidy in gliomas^11^. Longitudinal analysis of the somatic SNVs/INDELs in all three specimens revealed a monoclonal cancer formation model (Fig. 2b), where throughout disease progression a single clone was preserved; driven by a stop-gain mutation on *STAG2* gene (p.K554X). *STAG2* encodes a subunit of a cohesin complex, and its targeted inactivation has been shown to cause aneuploidy through sister chromatid cohesion^5^. Further analysis of the clonal evolution of these three specimens, revealed a branching structure, where a clone harboring an activating *EGFR* (p. A289D) mutation overlapping with chromosome 7 amplification was lost during disease progression. Specimen from the second surgery also harbored a private clone composed of mutations not shared by other specimens, including *TP53* (p.R43H) and *PTPN11* (p.E69K). The loss of a clonal mutation cluster and emergence of new ones when comparing specimens from the first to second surgeries, depict how the tumor evolved under the pressure of treatment from first-line temozolomide and radiation, salvage therapy with lomustine and temozolomide, and lastly with the recombinant poliovirus therapy. Interestingly, the specimen from the last surgery had a new emerging clone, harboring the oncogenic *BRAF* (D594G) mutation, which is categorized as class III *BRAF* mutation with impaired kinase activity and dependency to RAS activation^9^. Even though this mutation was subclonal, this indicated the tumor was still evolving in addition to preserving the *STAG2* mutation present in the founding clone.

We also performed mutational signature analysis on somatic SNV and INDEL data and investigated the enrichment of well-established cancer signatures^12^. Based on this analysis, signature-1 was the most aberrant signature across all specimens as expected, which has been seen across all cancer types (Fig. 2c, d). However, interestingly this analysis clustered specimens from the second and fourth surgeries together, consistent with the clonality analysis, showing that these tumors genomically diverged from the first surgery specimen. Signature 3 was the second most frequent signature in specimens from the second and fourth surgeries (Fig. 2c, d). Signature 3 has been shown to correlate with defects in DNA double-strand break (DSB) repair by homologous recombination (HR), and reported frequently in *BRCA*-mutated breast, ovarian, and pancreatic cancers^12^. This finding was consistent with the clonal evolution of the somatic mutations, as with the loss of a major clone harboring *EGFR*, the major oncogenic clone in specimens from the second and fourth surgeries still harbored the *STAG2* mutation, whose normal function in addition to maintaining sister chromatid cohesion, involves localization to DNA DSBs to repress aberrant transcription^13,14^, as well as signaling and recruitment of DNA damage checkpoint proteins^15–18^.

To further investigate the prevalence of *STAG2* mutations in glioblastoma, we investigated the TCGA database, which revealed *STAG2* as one of the 71 significantly mutated genes in 291 adult cases^19^. In a recent study of 326 pediatric high-grade gliomas, there were 3 cases with *STAG2* alterations, only one of which was a truncating mutation (<1%)^20^. We also investigated the current TCGA PanCancer Atlas cohort, where we identified 18 (14 truncating) *STAG2* mutant cases, out of 355 IDH1 wild-type glioblastomas (5.3%). Subsequently, we performed mutational signature analysis on these cases to compare to the COSMIC mutation signature distribution and observed that the most dominant signature in almost all adult cases was signature 1, with only one case showing a dominant signature-3; similar to the distinction between the primary tumor and two recurrences in the presented case (Supplementary Fig. 4).

## Discussion

Like in the adult population, treatment for recurrent pediatric glioblastoma remains limited and as such, our patient underwent multiple forms of medical therapy and re-irradiation prior to his passing. Specimens acquired from his multiple surgeries allowed us to characterize the genetic evolution of his tumor, including in response to certain treatments. Between his first and second surgeries, we observed near abolishment of the *EGFR*-mutated and amplified clone that was present in the original tumor. It is not possible to attribute the loss of this tumor clone to any particular therapy, as our patient underwent initial temozolomide and radiation, followed by lomustine and temozolomide, and lastly delivery of the recombinant poliovirus, all between his first and second surgeries. With subsequent resections, new tumor clonal populations emerged, harboring pathogenic loss-of-function mutations in *TP53* and *PTPN11*, and later, a key mutation in the oncogene, *BRAF*. However, through multiple recurrences, a key mutation in *STAG2* that was present in the initial specimen, persisted despite multimodal therapies, raising the possibility that the *STAG2* mutation was an early mutagenic event with potential tumor-driving activity.

*STAG2* encodes a protein integral to the cohesin complex, which has a key role in sister chromatid cohesion during mitosis^21,22^. Although there has been little correlation between aneuploidy and STAG2 deficiency in naturally occurring human tumors^23^, defects in the cohesin complex secondary to STAG2 inactivation has been shown to cause aneuploidy in human cancer lines^5^. In normal cells, STAG2 inactivation leads to stalling and collapse of replication forks, subsequent DNA DSBs, followed by checkpoint activation and cellular senescence. In STAG2 deficient tumor cells, the incurred DNA DSBs may promote further mutagenesis and tumor formation, particularly in TP53 deficient cells in which checkpoint activation is avoided^6^. STAG2 mutations have been reported in a variety of cancers, most frequently with robust preclinical and clinical data in Ewing sarcoma and urothelial carcinoma^24,25^. Within glioblastoma, approximately 4-6% of tumors harbor STAG2 mutations, according to large-scale genomic databases such as TCGA and COSMIC^19,26^. To our knowledge, our case is the first reported case of STAG2 mutation in pediatric glioblastoma. We hypothesize the *STAG2* mutation was key in promoting a defective DNA damage repair phenotype, contributing to further genetic changes and emergence of new tumor clones. This was corroborated by our mutational signature analysis, which revealed development of a cancer signature, characterized by DNA damage repair defects, and typically associated with *BRCA* mutations. Additional mutational signature analysis of 18 samples from the TCGA cohort revealed a predominance of signature 1 with only one case showing a dominant signature 3. However, these samples were all obtained at time of initial resection, raising the possibility that after standard chemoradiation and as observed in our patient, emerging tumor clones may exhibit greater DNA damage repair defects, consistent with mutational signature 3. However, it should be noted that there are some limitations to the mutational signature analysis, as only a few of them are suggestive of etiology or biological mechanisms. Having said that, for the signatures that are consistently shown to be related to certain mechanisms, such as signature 3 being attributed to DNA double strand break repair by HR, this analysis provides supportive data to show the impact of a proposed mechanism on mutation profiles. Further genetic characterization of recurrent *STAG2* deficient glioblastomas is needed to answer this question.

While the clinical significance of STAG2 deficiency in human cancers remains under investigation, preclinical data have shown a synthetic lethality conferred by STAG2 mutations with DNA DSB repair genes, including increased sensitivity to PARP and ATR inhibitors^6,27^. This has been previously studied in other biomarkers of defective DNA damage repair, including *IDH1/2* mutational status and *MGMT* promoter methylation^28–30^. On the other hand, STAG2 deficient cancer cells have also been shown to exhibit increased sensitivity in vitro to ionizing radiation and traditional chemotherapeutic agents, including temozolomide^6^, which contrasts with the aggressive clinical course of our patient. Perhaps, enhanced mutagenesis secondary to an unstable genome from STAG2 deficiency may have led to additional mutations promoting resistance to standard chemoradiation, highlighting the complexities in translating preclinical studies to clinical practice. Further data demonstrating exploitation of defective DSB repair with targeted DNA damage repair inhibitors may lead to similar clinical studies in *STAG2* defective tumors and also delineate mechanisms of resistance to standard chemoradiation in STAG2 deficient glioblastoma.

Immunotherapies have emerged in glioma treatment as a promising therapeutic approach for advanced, progressive disease, including use of the recombinant poliovirus, as seen in our patient. Desjardins et al. published their results of a dose-finding and toxicity study for adult patients with recurrent glioblastoma who underwent convection-enhanced, intratumoral delivery of the recombinant nonpathogenic polio-rhinovirus chimera^31^, which recognizes the poliovirus receptor CD155 that is expressed in many different tumors, including glioblastoma^32,33^. Over a 5 year period, 61 patients were enrolled in the study, and the treatment was generally well-tolerated with the majority of patients experiencing only grade 1 or grade 2 events as their most severe adverse event and no evidence of viral neuropathogenicity or virus shedding. Median overall survival among patients receiving recombinant poliovirus was 12.5 months, compared to 11.3 months in the historical control group. Furthermore, survival at 24 and 36 months plateaued at 21% in patients treated with the recombinant poliovirus, compared to a progressive decline of 14% and 4% in the historical controls. Currently, there is an ongoing phase 2 randomized trial of the recombinant poliovirus alone or in combination with lomustine in patients with recurrent glioblastoma (NCT02986178). Although our patient was not enrolled in the original clinical trial, given his pediatric age, an additional novelty of this report lies in the genetic characterization of tumor changes after administration of the recombinant poliovirus therapy. However, considering other chemotherapies and radiation were also implemented between the first and second surgeries, it is not possible to attribute the changes in the genetic landscape of the tumor at time of first recurrence to the poliovirus treatment alone. Additionally, the investigation of the tumoral response to the recombinant poliovirus was limited only to routine immunohistochemistry for standard immune markers. A more in-depth analysis such as sequencing of the immune infiltrate may have provided further insight into the patient’s response to immunotherapy. Taken together, further studies are needed to delineate the clonal populations within glioblastoma that may resist recombinant poliovirus therapy and lead to clinical progression.

In addition to emergence of the aforementioned subclonal populations after chemoradiation and recombinant poliovirus therapy, a third distinct tumor clone developed, present in the resected specimen from the fourth surgery and defined by the *BRAF* D594G mutation. *BRAF* is a known oncogene, whose mutation most frequently at the V600 hotspot drives constitutive activation of the MAP kinase/ERK signaling pathway, and has been well-characterized in multiple cancers including melanoma, thyroid cancer, lung cancer and brain tumors including glioblastoma and pleomorphic xanthoastrocytoma^34^. Mutations at the D594 site are far less common, rarely reported in melanoma^35^ but more frequently in lung and colorectal cancers^9,36^. Unlike the V600 mutation, the D594 mutation, one of oncogenic class III *BRAF* mutations, is kinase-impairing and may drive ERK signaling through a complex with CRAF leading to hyperactivation of the CRAF/MEK/ERK cascade and may subsequently be more sensitive to MEK inhibitors^37^. Within pediatric gliomas, *BRAF* mutations have only been reported at the V600 hotspot^38–40^ whose therapeutic inhibition has been studied in clinical trials with varying degrees of efficacy^41,42^. As such, this case represents the first report of a non-V600 BRAF mutation in pediatric glioma. While this clone was only detected after the patient’s fourth surgery, which included re-irriadiation after recombinant poliovirus administration, it is plausible that the clone may have existed in lower numbers at the time of second and third surgeries, at levels too low to detect with routine WES. Therapeutically, targeting the kinase-dead V594 mutant BRAF mutation may not be effective with direct BRAF inhibitors; however it is reported that cases with class III BRAF mutations may be sensitive to MEK inhibitors that inhibit downstream signaling^37^ and also to combination therapies with EGFR inhibitors targeting the RAS dependency^9^. Interestingly, RAS dependency in class III *BRAF* mutated lung and colorectal cancers required activation of receptor tyrosine kinases^9^, which may have been the case in our patient where receptor tyrosine kinase activation was mediated through the *EGFR* mutation and amplification observed in the specimen from the first surgery. Although the *BRAF* mutation was not detected until later on in the clinical course, it is plausible that the *BRAF* mutated clone may have existed in lower numbers at earlier resections at levels too low to detect with routine WES. Further studies are needed to determine the clinical significance of non-V600 mutations in pediatric glioma and their potential for therapeutic targeting.

The striking clonal divergence among these three tumors with eradication and emergence of clinically significant sub clones is perceived from the temporal dimension by three distinct time point sampling. However, we should emphasize that our understanding of this complex evolution of the tumor would have benefitted immensely by the spatial data as well. However, due to clinical urgencies surrounding the patient’s subsequent surgeries, there was only limited specimen available only from a single sector of the tumor, limiting the possibility of the multiregion analysis.

In summary, we report the genetic alterations of a *STAG2* deficient pediatric glioblastoma after undergoing first-line temozolomide and radiation, salvage lomustine and temozolomide, recombinant poliovirus treatment, and lastly re-irradiation. The *STAG2* mutation was persistent across all specimens, signifying a potential key role for *STAG2* deficiency in gliomagenesis, including a role in promotion of further genetic alterations that may drive tumor progression. In our patient, this included development of a non-V600, D594G *BRAF* mutation, previously uncharacterized in pediatric glioma, whose kinase-activating properties may be sensitive to MEK inhibition. Additionally, we found a striking elimination of the *EGFR*-mutated and amplified tumor clone after first-line chemoradiation followed by novel recombinant poliovirus therapy and subsequent emergence of a new clonal population, characterized by a DNA damage repair defect signature. With increasing preclinical evidence of *STAG2* deficiency playing a key role in tumor formation, further genetic characterization of *STAG2* mutated gliomas after standard-of-care surgical and medical therapies are needed to understand the genetic evolution of these tumors and potential mechanisms of resistance. This study also emphasizes the importance of comparative genomic characterization throughout the disease progression, to better understand the drivers and how they evolve especially in highly heterogenous tumors such as glioblastoma.

## Methods

### Patient approval

This study was approved by Yale University’s Human Investigations Committee and Human Research Protection Program. Written informed consent was obtained from the parents of the patient. Specimens from resected tumors were evaluated microscopically by a board-certified neuropathologist.

### Whole exome sequencing and analysis

Genomic DNA from the resected tumor specimens and blood were isolated, and exome capture was performed with IDT xGen Exome Research Panel v1 with the additional spike-in of ~2500 regions totaling ~620 kb of RefGene coding regions. Sequencing was performed at Yale Center for Genome Analysis (YCGA) using Illumina NovaSeq6000 with 2×100 bp reads. We achieved mean coverage of 207×, 286.7×, and 306.9× for tumors from surgery 1, surgery 2, and surgery 4, respectively. Mean coverage of 133.6× was achieved for matching blood (Supplementary Table 1). Processing of the raw reads, alignment, polymerase chain reaction duplicate identification, re-alignment, base quality score recalibration, somatic variant calling and annotation was performed as previously described in reports from our institution^43^. For SNV/INDELs, matching normal data was used to perform somatic analysis. Copy number alterations were identified following the guidelines in the Genome Analysis Toolkit (GATK) Best Practices. For Somatic CNV identification, we have utilized the panel of normal controls from the Yale Brain Tumor WES cohort, instead of just using the corresponding matching normal data. Mutation signature analysis for both the patient data and the TCGA data was performed using MutationalPatterns R package^44^. Clonality analysis on the somatic SNV/INDELs for all three specimens was performed using ClonEvol package^45^ to calculate the cellular fraction in a CNV sensitive manner, to identify clones, and to infer the tumor phylogeny. Visualization of the inferred phylogeny was performed using fishplot R package^46^.

### Reporting summary

Further information on research design is available in the Nature Research Reporting Summary linked to this article.

## Supplementary information

Supplementary Information

Reporting Summary

## Data Availability

The somatic variant call files (vcf) for all three tumors are submitted to European Genome-phenome Archive (EGA) with accession number EGAS00001004340.
