# Supplementary material for: Persistent STAG2 mutation despite multimodal therapy in recurrent pediatric glioblastoma
Source: NPJ Genom Med. 2020 Jun 1;5:23. doi: 10.1038/s41525-020-0130-7 (PMC7264170; doi:10.1038/s41525-020-0130-7)
Supplement: Supplementary file 1 — Supplementary Information [file 41525_2020_130_MOESM1_ESM.pdf]

SUPPLEMENTARY INFORMATION

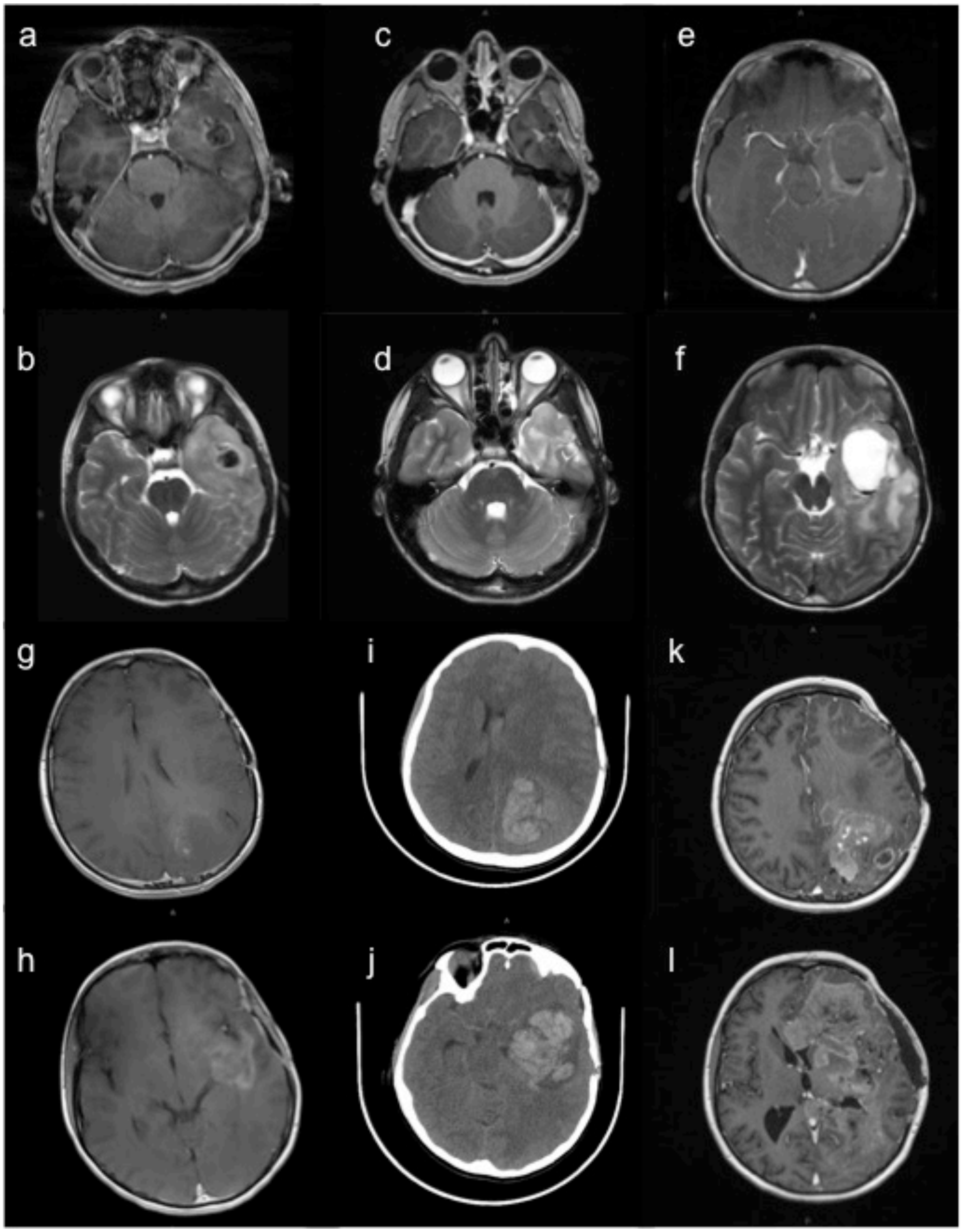

Supplementary Figure 1. Relevant imaging throughout clinical course. (a) T1-weighted MRI post-contrast and (b) T2-weighted MRI demonstrate a contrast-enhancing left temporal lesion at time of initial presentation with surrounding edema. After gross total resection, followed by adjuvant chemoradiation and an additional chemotherapy regimen, there was new nodular enhancement on (c) T1-weighted MRI post-contrast with surrounding edema on (d) T2-weighted MRI, concerning for disease recurrence. Three months after undergoing convection-enhanced delivery of the recombinant poliovirus, there was evidence of tumor recurrence on (e) T1-weighted MRI post-contrast with (f) T2-weighted MRI showing a prominent cystic component. (g) T1-weighted MRI post-contrast showed a new area of enhancement within the left occipital lobe, as well as (h) recurrent disease within the original tumor location. An emergent head CT obtained after the patient presented unresponsive showed acute hemorrhage within the (i) occipital and (j) temporal tumors. T1-weighted MRI post-contrast at time of last surveillance imaging prior to hospice care showed significant tumor progression within the (k) occipital lobe, as well as (l) new extension of the temporal focus across the corpus callosum into the contralateral hemisphere.

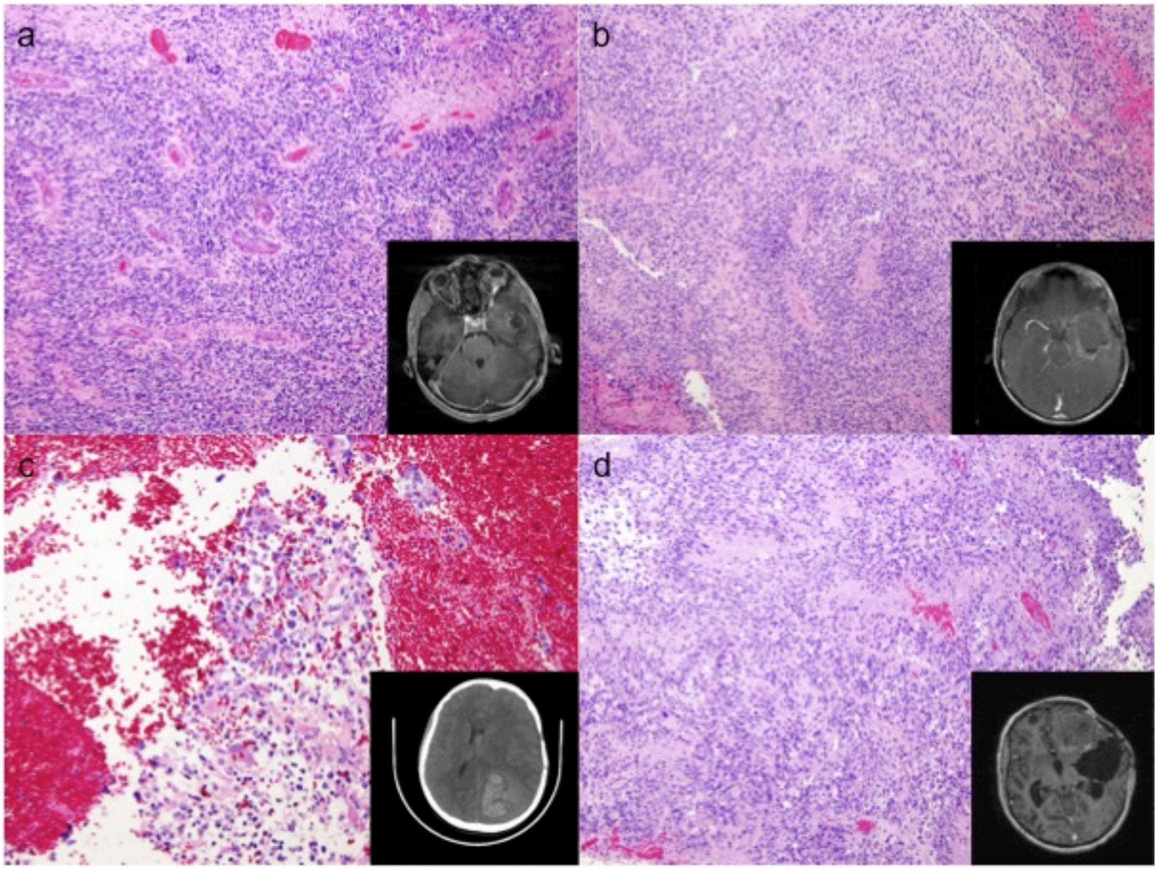

Supplementary Figure 2. Hematoxylin & Eosin stained sections of tumor tissue obtained during surgical resections with representative inlayed pre-operative imaging demonstrate classic histomorphologic features of glioblastoma, such as high cell density, pleomorphism, numerous mitoses, microvascular ("endothelial") proliferation and palisading necrosis in the (a) first (b) second and (d) fourth resection specimen. (c) The third resection revealed mainly hemorrhage with only minute fragments of intermixed glioblastoma.

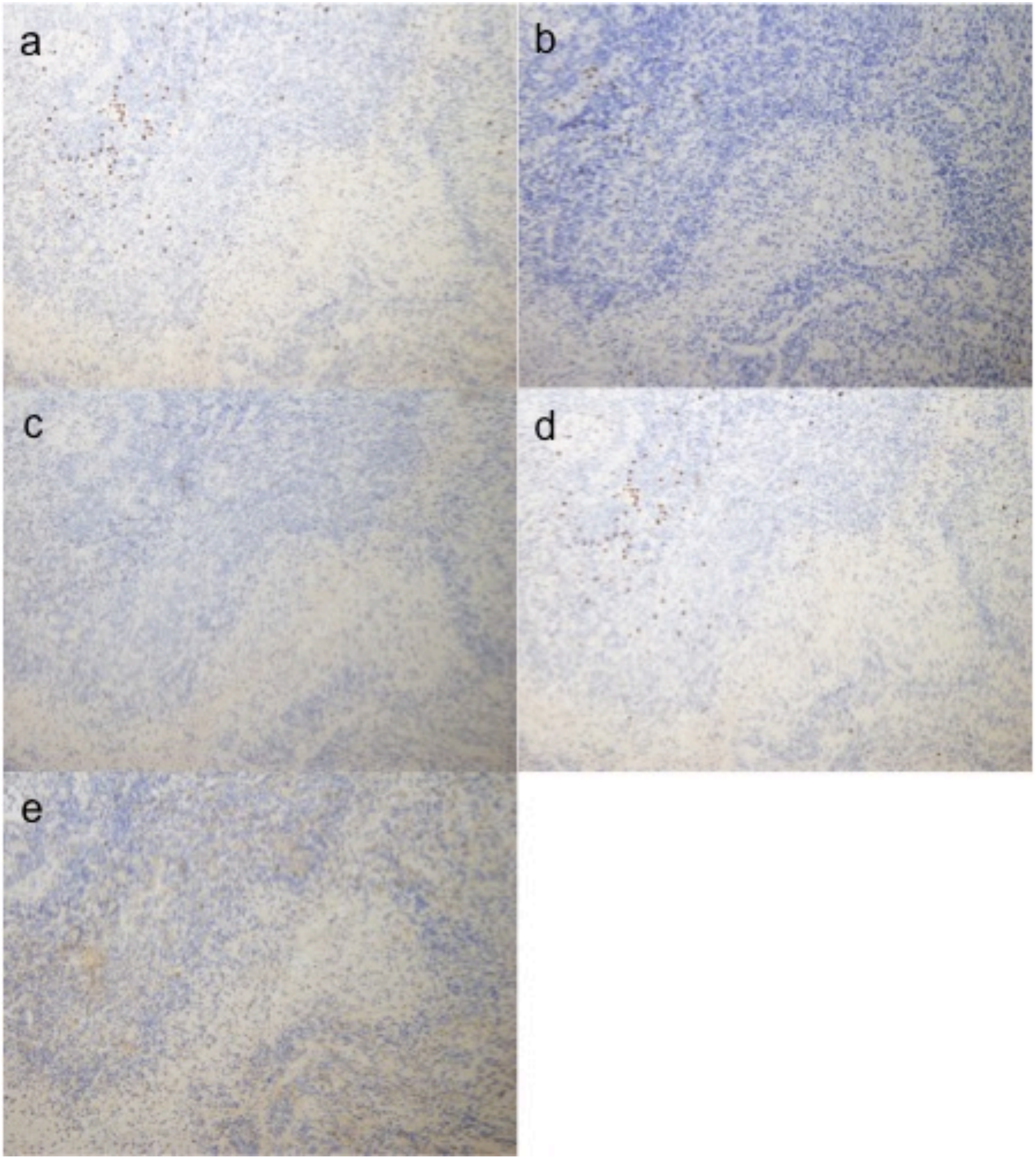

Supplementary Figure 3. Immunohistochemical stains from the second surgery for (a) CD3 and (b) CD8 show only few intermixed intraparenchymal T-lymphocytes and (c) very scant CD20 positive B-lymphocytes. (d) CD163 positive macrophages are seen in abundance around the edges of necrosis but also within tumor parenchyma. (e) PDL1 expression is negative.

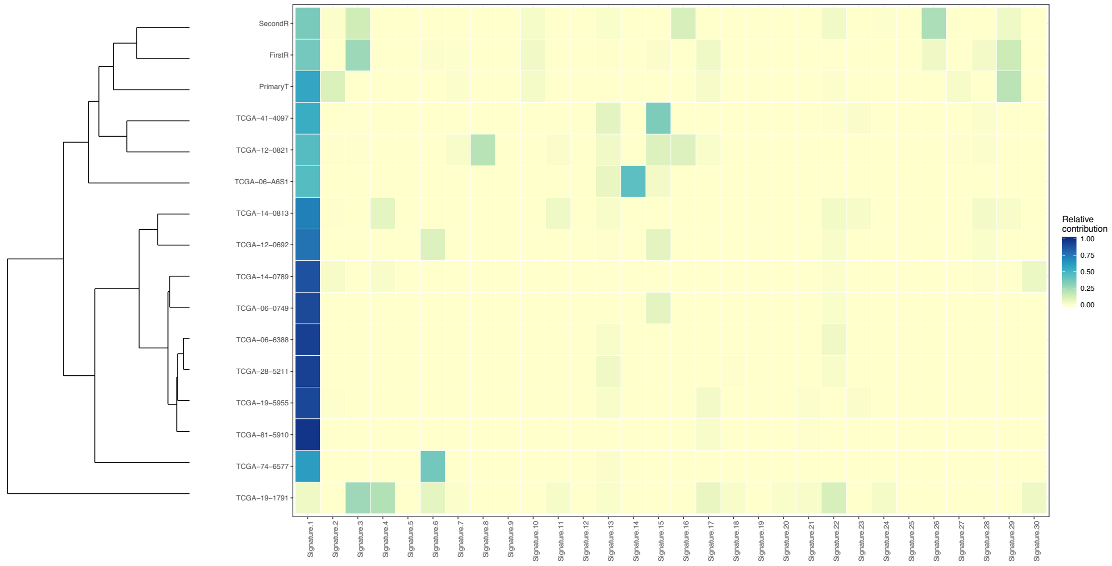

Supplementary Figure 4. Clustering of cosine similarity matrix of 96 mutation signatures for the presented cases together with the STAG2 mutant TCGA cases and the 30-COSMIC signatures. Complete agglomeration method is used to cluster samples with Euclidean distances of the cosine similarity values using MutationalPatterns package in R.

Specimen Source	Read Length	Mean Coverage	20X Target Base Coverage	30X Target Base Coverage	Purity
Tumor Surgery 1	101	207.1	95.20%	94.90%	98.50%
Tumor Surgery 2	101	286.7	95.20%	95.10%	97.50%
Tumor Surgery 4	101	305.9	98.80%	98.70%	73.80%
Matching Normal Blood	101	154.2	98.80%	98.50%	NA

Supplementary Table 1. Whole-exome sequencing metrics.
